# Comprehensive Quality Evaluation of *Polygonatum cyrtonema* and Its Processed Product: Chemical Fingerprinting, Determination and Bioactivity

**DOI:** 10.3390/molecules28114341

**Published:** 2023-05-25

**Authors:** Jianguang Zhang, Junjun Wang, Li Yang, Yue Wang, Wenfang Jin, Jing Li, Zhifeng Zhang

**Affiliations:** 1 Tibetan Plateau Ethnic Medicinal Resources Protection and Utilization Key Laboratory of National Ethnic Affairs Commission of the People’s Republic of China, Southwest Minzu University, Chengdu 610041, China; zhangjg0777@sina.com (J.Z.); 18308211183@163.com (L.Y.); yuewang724@163.com (Y.W.); jingli3485@163.com (J.L.); 2 Qin Zhou Provincial Health School, Qinzhou 535009, China

**Keywords:** *Polygonatum cyrtonema* Hua, UPLC-Q-Exactive-MS/MS, GC-MS, processing, bioactivities

## Abstract

Processing of Chinese herbal medicines (CHMs) is a traditional pharmaceutical technology in Chinese medicine. Traditionally, proper processing of CHMs is necessary to meet the specific clinical requirements of different syndromes. Processing with black bean juice is considered one of the most important techniques in traditional Chinese pharmaceutical technology. Despite the long-standing practice of processing *Polygonatum cyrtonema* Hua (PCH), there is little research on the changes in chemical constituents and bioactivity before and after processing. This study investigated the influence of black bean juice processing on the chemical composition and bioactivity of PCH. The results revealed significant changes in both composition and contents during processing. Saccharide and saponin content significantly increased after processing. Moreover, the processed samples exhibited considerably stronger DPPH and ABTS radical scavenging capacity, as well as FRAP-reducing capacity, compared to the raw samples. The IC_50_ values for DPPH were 1.0 ± 0.12 mg/mL and 0.65 ± 0.10 mg/mL for the raw and processed samples, respectively. For ABTS, the IC_50_ values were 0.65 ± 0.07 mg/mL and 0.25 ± 0.04 mg/mL, respectively. Additionally, the processed sample demonstrated significantly higher inhibitory activity against α-glucosidase and α-amylase (IC_50_ = 1.29 ± 0.12 mg/mL and 0.48 ± 0.04 mg/mL) compared to the raw sample (IC_50_ = 5.58 ± 0.22 mg/mL and 0.80 ± 0.09 mg/mL). These findings underscore the significance of black bean processing in enhancing the properties of PCH and lay the foundation for its further development as a functional food. The study elucidates the role of black bean processing in PCH and offers valuable insights for its application.

## 1. Introduction

*Polygonatum cyrtonema* Hua (PCH) belongs to the genus Polygonatum (Asparagaceae), which is mainly distributed in the North Temperate zones of Asia, such as India, Afghanistan, Pakistan, Korea, China and Japan [1]. The plant is also widely planted in Sichuan, Guizhou, Hunan, Jiangxi, and other regions in China. The rhizomes of PCH, called “Huangjing” in China, have been widely used as a functional food and herbal medicine [2]. With the function of invigorating Qi, nourishing Yin and moistening the lungs, it is often used to replenish energy, strengthen immunity and treat fatigue, weakness, diabetes and lung disorders [1]. Modern pharmacological studies have shown that PCH exhibits antioxidant [3], antidiabetic [4], antitumour [5], anti-inflammatory [6] and anti-osteoporotic effects [1]. Multiple components of PCH can interact in a synergistic fashion to potentiate its pharmacological activity. The open innovation platform such as International Natural Product Sciences Taskforce could help researchers to easily identify the major constituent(s) of PCH’s exerting activity [7].

In China, PCH is used after processing, because processed PCH exhibits better effects than the raw [1]. Generally, the raw rhizomes of PCH contain mucilaginous ingredients, and they are rarely taken orally directly due to its stimulating tongue. Therefore, the raw must be processed to remove these ingredients and enhance its tonic function. Depending on the theories of traditional Chinese medicine, PCH is processed by steaming using wine, black soybean, ginger and honey before clinical applications [8]. The most common traditional processing method is to steam and sun-dry the rhizomes of PCH nine times until the rhizomes turn black, soft and sweet. Numerous studies have indicated that some chemical compositions are increased or decreased during processing, and these variations might have a significant effect on biological activities [3,9,10]. Processing with black beans is a specific pre-treatment for many Chinese herbs before drying, which is usually applied to Chinese herbs, including toxic or irritating components, which is also beneficial in enhancing the efficacy of nourishing the liver and kidney and to reduce the toxicity and side effects of herbs [11,12]. Processing of PCH with black beans is a method recorded in the Processing Standard of Chinese Herbal Pieces in Sichuan Province 2015 Edition [13], while fewer studies have reported processing with black beans in rhizomes of PCH. Moreover, no studies have reported the differences in chemical composition and bioactivities among the raw and processed black beans of PCH. There is a lack of credible information to clarify the variations during PCH processing. Thus, it is meaningful to study the variations in chemical composition and bioactivities from processing with black beans in PCH.

Studies on phytochemicals have indicated that PCH contains many types of bioactive components, including polysaccharides [14], oligosaccharides [15], steroidal saponins [16], triterpenoid saponins [17], flavonoids [18] and so on. Polysaccharides are regarded as the main chemical constituents with various bioactivities, such as antioxidant protection and antidiabetic action. Polysaccharide content was regarded as an index for the quality control of PCH in the Chinese Pharmacopoeia 2020 Edition (Volume I) [19]. Concerning the analysis of small biological molecules, ultrahigh-performance liquid chromatography coupled with time-of-flight mass spectrometry (UHPLC-Q-TOF-MS/MS), with the advantages of rapid analysis time and high selectivity and sensitivity, is a powerful tool for detecting and analysing small biomolecules [20,21,22]. It is also a good analytical technique to screen differential compounds during processing [23,24]. Polysaccharides are macromolecules and saccharides with complex structures. Many saccharide test methods have been developed, including gas chromatography-mass spectrometry (GC-MS), high-performance anion exchange chromatography (HPAEC), liquid chromatography-mass spectrometry (LC-MS), gas chromatography (GC) and high-performance liquid chromatography-evaporative light scattering detection (ELSD). GC-MS has the advantages of high sensitivity, strong separation ability and accurate qualitative identification, and it has been widely used in the analysis of saccharides [25,26]. Chemometrics analysis technology, combining mathematics and statistics, can objectively analyse complicated data, and it has been applied to the quality control of traditional Chinese medicine [27,28,29].

The objectives of this study were to investigate of the differences in chemical components and biological activity between a raw sample and a black bean processed sample of PCH; UHPLC-Q-Exactive-MS/MS was used to identify the steroidal saponins, triterpenoid saponins, flavonoids and so on. The polysaccharides and saponin contents were determined by UV. GC-QQQ-MS/MS was used to quantify the monosaccharide composition of PCH. The bioactivities of the raw and the processed PCH were evaluated in vitro by antioxidant and antidiabetic assays. This study aims to elucidate the physicochemical properties and activity regulation of polysaccharides and saponins present in the active ingredients of black bean-processed PCH and investigate the scientific implications of antioxidant and hypoglycaemic activities through the modulation of these polysaccharides and saponins. Furthermore, the study’s results may provide valuable insights into the development of novel foods and functional products utilizing polysaccharides and saponins from black bean processed PCH.

## 2. Results and Discussion

### 2.1. Method Optimization

**Figure 1:**
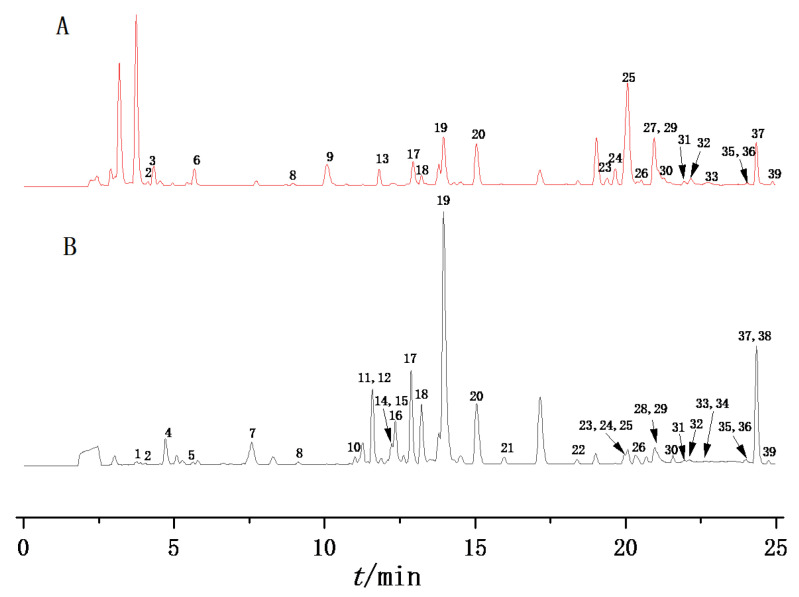
The TIC of processed PCH (**A**) and raw PCH (**B**).

To obtain satisfactory separation and more peak pattern, the influence of mobile phase (water-acetonitrile, water-methanol, 0.1% aqueous formic acid-acetonitrile and 0.1% aqueous formic acid-methanol), column type, flow rate (0.2, 0.3 and 0.5 mL/min), detection wavelength (210, 254 and 280 nm) and column temperature (25, 30 and 35 °C) were evaluated. As a result, a Hypersil GOLD C18 column (2.1 × 100 mm, 1.8 μm) was chosen, with the best chromatographic separation of samples. The mixture of 0.1% aqueous formic acid (A)-acetonitrile (B) was used for the mobile phase, with its better peak shapes, better resolution and higher response values. The column temperature was set at 30 °C, and the flow rate was 0.3 mL/min. As shown in Figure 1, 39 peaks of the samples were separated and detected within 25 min.

### 2.2. Identification of Chemical Compounds by UPLC-Q-Exactive-MS/MS

In this work, the chemical components of raw and processed PCH samples were detected and identified by UPLC-Q-Exactive-MS/MS in both positive and negative ion modes. The positive ion showed a preferable detection effect with the peak purity and number of detected peaks. The total ion currents (TICs) of the raw and processed PCH samples are shown in Figure 1. In total, 39 compounds were detected, and 33 of them were identified, including flavonoids, alkaloids, coumarins, fatty acids, benzene, substituted derivatives and other compounds. Of these, compounds **9** and **19** were unambiguously identified by comparison with reference standards. The detailed information of these compounds is displayed in Table 1, including fragment ions, retention time, molecular formula and so on.

**Figure 2:**
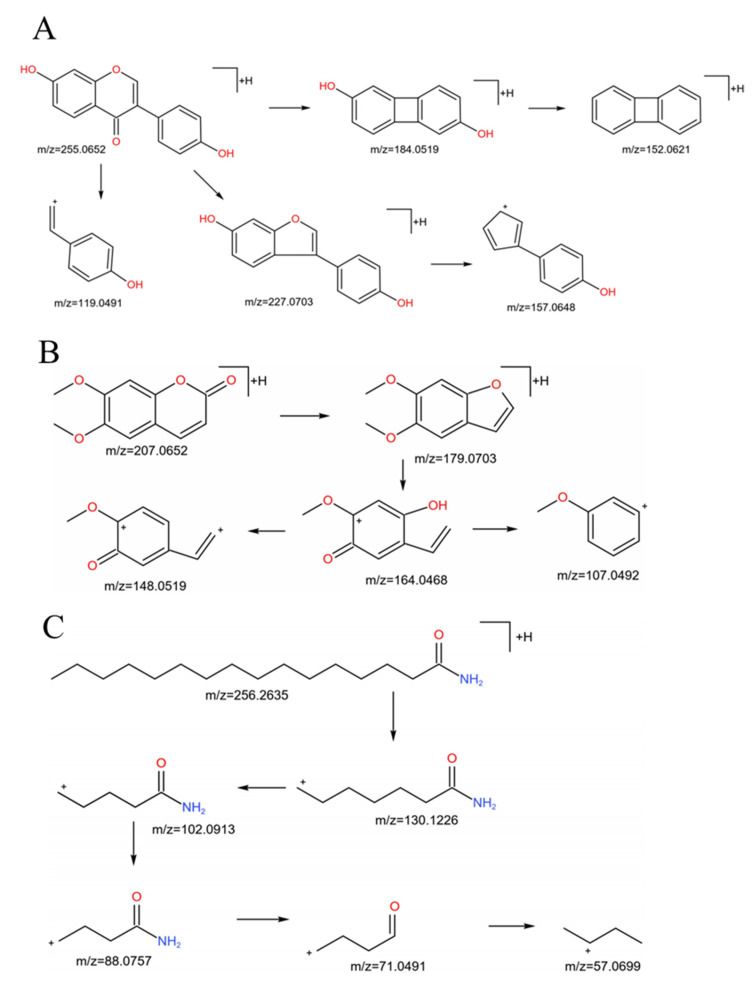
The hypothesized fragmentation pathway of compound **9** (**A**), compound **19** (**B**) and compound **39** (**C**).

Compounds were identified according to their characteristic fragment ions compared to reported references and standards. For example, compound **9** (flavonoid) showed an [M + H]^+^ ion at *m*/*z* 255.06584 (C_15_H_10_O_4_) and exhibited fragment ions at *m*/*z* 227.07100 by the losses of CO. It also produced fragment ions at 137.0230 (1.3A^+^) and 119.04977 by fragmentation pathways of Retro-Diels-Alder (RDA). It can be definitively confirmed to be daidzein through comparison with the literature and reference standard. The hypothesized fragmentation process of compound **9** is displayed in Figure 2A. Compound **19** (coumarin) yielded an [M + H]^+^ ion at *m*/*z* 207.06589 with t formula C_11_H_10_O_4_, and then it lost a molecule of CO, CH_3_ and H_2_O to form a [M + H − CO]^+^ fragment ion of *m*/*z* 17907032, [M + H − CO − CH_3_]^+^ fragment ion of *m*/*z* 164.04680 and [M + H − CO − CH_3_ − H_2_O]^+^ fragment ion of *m*/*z* 148.05188. Compound **19** was unambiguously confirmed as scoparone through comparison with the literature and standard substance. The hypothesized cleavage mode of compound **19** is shown in Figure 2B. Compound **39** displayed an [M + H]^+^ ion at *m*/*z* 256.26447 with the formula C_16_H_33_NO, and it exhibited characteristic fragment ions at *m*/*z* 130.12366, 102.09198, and 88.07630, indicating successive losses of C_9_H_18_, C_2_H_4_, and CH_2_. Compound **39** was tentatively inferred to be hexadecanamide, and the hypothesized fragmentation pattern is shown in Figure 2C.

### 2.3. Comparison of Chemical Profiles of Raw and Processed PCH

The newly established UPLC-Q-Exactive-MS/MS method was used to qualitatively and semi-quantitatively compare the chemical fingerprints between raw and processed PCH samples. As shown in Figure 1, it was notable that the quantity and intensity of lower polarity compounds increased significantly in processed samples, while the medium polarity compounds clearly decreased and even disappeared after processing. There were six newly generated compounds (peaks 3, 6, 9, 13, 24 and 27) in processed PCH samples. The intensities of peaks 25 and 29 were markedly increased in processed PCH samples, while peaks 17, 18, 19, 28 and 37 were sharply decreased. Peaks 7, 10, 12, 14,15, 16, 21, 22, 28 and 38 of processed samples disappeared completely. The results indicated that processing could have caused some chemical changes in PCH during processing with black beans, which consequently changed the chemical fingerprints of the PCH samples.

### 2.4. Multivariate Statistical Analysis and Discovering Potential Chemical Markers

**Figure 3:**
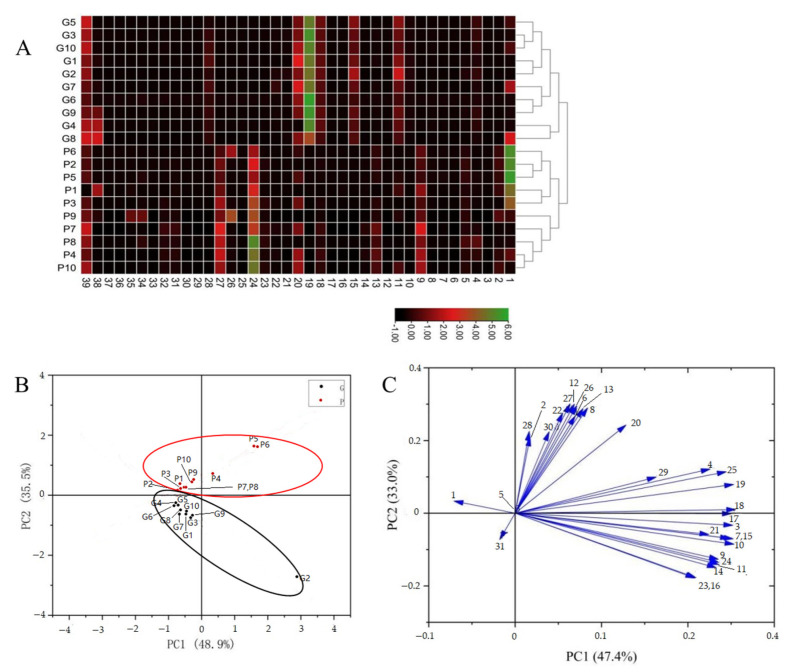
The cluster heat map (**A**), the PCA score (**B**) and the loading plot of PCA in raw and processed PCH (**C**).

Due to chemical complexity, the differences between raw and processed PCH samples were unclear according to UHPLC-Q-Orbitrap-MS chromatograms. To distinguish the raw and processed PCH samples, a cluster heatmap was first carried out using TBtools software (TIBCO Software Inc., Palo Alto, CA, USA). In the cluster heatmap, different colors represent different content, with bright green representing higher content and dark red representing lower content. As shown in Figure 3A, the results of the cluster heatmap showed that all the samples were distributed into two major clusters. One cluster was regarded as the raw samples (G1–G10), and another cluster was considered to be the processed samples (P1–P10). It should be noted that the content of these compounds (peaks 1, 9, 11, 13, 15, 18, 19, 20, 24, 27, 28 and 39) are more than other compounds, and they may be the key compounds of differences between raw and processed PCH samples, suggesting that the chemical component and/or contents of compounds may change remarkably in the procedure of processing with black beans.

To further demonstrate the differences between the raw and processed PCH samples, unsupervised principal component analysis (PCA) statistical analysis, taking into account all variables, was further employed to analyze the MS data using Origin Pro 2022 software (Microcal Software, Northampton, MA, USA). The first and second principal components explained 48.9% and 35.5% of the total variance, respectively, meaning that the analytical platform was stable. The scatter plots of PCA indicated that all the samples were divided quite obviously into raw and processed PCH groups (Figure 3B). The raw samples (G1–G10) were distributed below the plot, while the processed samples (P1–P10) were at the upper of the plot, suggesting that the procedures of processing with black beans induced remarkable changes in the chemical component and/or contents of compounds in PCH. The loading plot of PCA is shown in Figure 3C. The spot will be far from the original point, meaning that it contributes the most to the differences between raw and processed PCH samples. Thus, these peaks had a significant contribution to showing the differences between raw and processed PCH samples except peaks 1, 5 and 31 because they were far away from the center in the model, and these compounds could be considered as the different markers for the raw and processed PCH samples. Therefore, these results demonstrated great differences in chemical constituents between the raw and processed PCH.

### 2.5. Polysaccharides Content of PCH

In this study, glucose was used as a standard. The equation of the regression line was *y* = 28.37*x* + 0.0011 with r = 0.9997, and the polysaccharide content was obtained from a regression line of glucose in the 2–16 μg/mL range, showing good linearity. The RSD values of the precision and repeatability tests were within 3.0% and 2.3%, respectively. The RSD values of the stability were no more than 2.8% within 12 h post reaction. The recovery rate of glucose was 103.0%, and the RSD was within 3.4%, indicating an accurate and reliable method. As listed in Table 2, the polysaccharide content of raw samples ranged from 8.46% to 14.88%, and the average value of polysaccharide content was 12.19%. However, the content of polysaccharides in processed samples ranged from 29.40% to 35.12%, and the average value of polysaccharide content was 32.28%. The results indicated that the processed samples suggested an obvious increase in polysaccharide content. The variation showed that processing with black beans has an obvious effect on polysaccharide content in PCH, which may induce some compounds with glycoside structures to degrade to oligosaccharides, consistent with earlier studies [30,31].

### 2.6. Saponin Content of PCH

Saponins are also the main bioactive compounds in PCH [24]. In this study, diosgenin was used as a standard. The equation of the regression line was *y* = 38.693*x* − 0.1676 with r = 0.9998, and the saponin content was calculated by a standard curve of diosgenin ranging between 6 and 16 μg/mL. The RSD values of the precision and repeatability tests were less than 2.1% and 2.6%, respectively. The RSD values of the stability were within 2.7% in 12 h. The recovery rate was 103.2%, and the RSD value was within 2.7%. These results showed that the approach could be suitable for total saponin content measure. As shown in Table 2, the saponin content of raw samples ranged between 3.64% and 8.50%, and the average value of saponin content was 5.01%. However, the saponin content of processed samples ranged from 6.09% to 9.41%, and the average value of saponin content was 7.69%. The results revealed that there were a few differences among the raw and processed samples, and processing caused a change in some chemical structures and showed a rising trend in saponin content.

### 2.7. Quantitative Analysis of Monosaccharide by GC-MS

#### 2.7.1. Method Validation

Method validation for the determination of monosaccharide results is listed in Table 3. The results of the linear equation, LOQs and LODs for the five monosaccharides showed a good linearity (r > 0.9990) for each reference standard within a certain concentration range. For the precision test, the intraday and inter-day RSDs were no more than 1.81% and 2.52%, respectively, which indicated that the instrument was in order. The RSD values of the repeatability were within 2.40%, and the established method was reproducible. The stability showed that the sample solution had a steady repetition at 24 h at room temperature, and the RSD values were less than 1.84%. Moreover, the average recoveries ranged from 94.47% to 103.23%, and the RSD values of recovery were no more than 1.39%. The results indicated that the newly established approach can be suitable for quantitative analysis.

#### 2.7.2. Monosaccharide Composition and Levels Differ between Raw and Processed PCH

**Figure 4:**
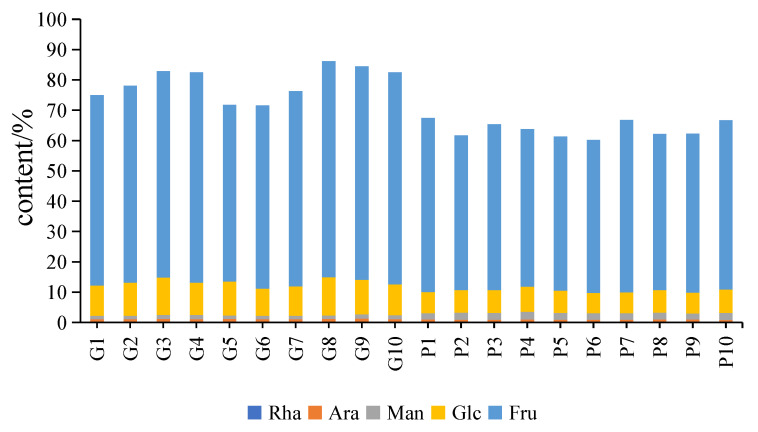
The content of monosaccharide in extracts of the raw (G) and processed (P) samples of PCH.

GC–MS was utilized to determine monosaccharide composition and levels in different PCH samples. The monosaccharide composition analysis revealed that the extracts of both raw and processed PCH samples primarily contained five kinds of monosaccharides, including rhamnose, arabinose, mannose, glucose and fructose, by comparison with the reference standards. The monosaccharide content hydrolyzed from the extracts is listed in Figure 4. After processing, the contents of arabinose, glucose and fructose hydrolyzed from extracts were 0.7114%, 7.3248% and 53.3072%, respectively, and were reduced by 0.25%, 3.46% and 12.70%, respectively, while the contents of rhamnose and mannose were 0.2161% and 2.2363%, respectively, and increased by 0.03% and 1.03%, respectively. Specifically, fructose was the most abundant monosaccharide hydrolyzed product in both raw and processed PCH. The monosaccharide composition and level results showed that processing had no obvious impact on the type of monosaccharides present in the extract but changed their content and ratio, which could further induce chemical structure variations, consistent with previous studies [2,24].

### 2.8. Antioxidant Activity Test Results

**Figure 5:**
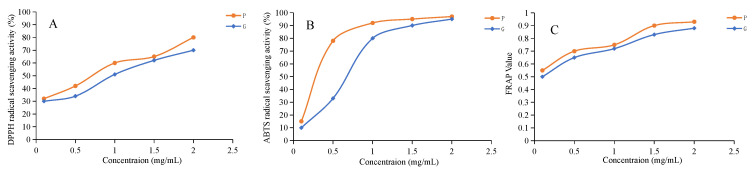
DPPH radical scavenging activity (**A**), ABTS radical scavenging activity (**B**) and Ferric reducing antioxidant power (**C**). G, the extract of the raw sample; P, the extract of processed sample.

To compare the antioxidant capacity of raw and processed samples, antioxidant assays were performed (Figure 5A–C). DPPH and ABTS are stable free radical commonly used to evaluate the total antioxidant capacity of antioxidant materials. DPPH and ABTS were therefore used to evaluate the radical scavenging activity of the raw and processed PCH. As shown in Figure 5A,B, the raw and processed samples both displayed DPPH and ABTS radical-scavenging activities in dose-dependent at concentrations from 0 to 2.0 mg/mL. In addition, the antioxidant capacity of the processed sample was significantly stronger than that of the raw sample (*p* < 0.05). The IC_50_ values of the raw and processed samples for DPPH were 1.0 ± 0.12 and 0.65 ± 0.10 mg/mL, respectively, while the IC_50_ values for ABTS were 0.65 ± 0.07 and 0.25 ± 0.04 mg/mL, respectively. We also determined the reducing activity of the raw and processed samples by using the FRAP assay (Figure 5C). Both the raw and processed samples also showed dose-dependent scavenging activity, and the processed samples showed a higher FRAP reducing capacities than the raw samples. These results indicated that the processing with black beans had an obvious influence on the antioxidant activity of PCH, and antioxidant activity of processing with black beans sample showed significantly stronger than that of the raw sample. These studies are consistent with recent studies [3,9,30] that showed that the antioxidant activity of PCH was enhanced during steam processing.

### 2.9. Antidiabetic Activity In Vitro

**Figure 6:**
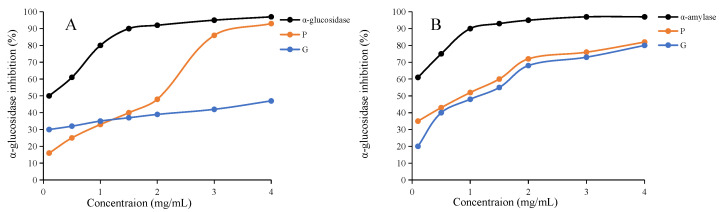
The α-glucosidase inhibitory activity (**A**) and the α-amylase inhibitory activity (**B**). G, the extract of the raw sample; P, the extract of processed sample.

In addition to antioxidant effects, PCH has also exhibited an antihyperglycemic effect in previous studies [3]. The inhibition activity of α-glucosidase and α-amylase activities of the raw and processed sample extracts was studied in this work. As shown in Figure 6A,B, the inhibitory effects of raw, processed and acarbose (positive control) on α-glucosidase and α-amylase activity showed an increase with a dose-dependent manner. As expected, acarbose showed the highest inhibition of α-glucosidase and α-amylase. The extracts of raw and processed PCH also showed significant inhibition against α-glucosidase and α-amylase. Lower IC_50_ values indicate stronger inhibition. The IC_50_ values of the extracts from raw and processed samples against α-glucosidase were 5.58 ± 0.22 and 1.29 ± 0.12 mg/mL (Figure 6A), respectively, and the extracts of raw and processed samples also had strong α-amylase inhibition with IC_50_ values of 0.80 ± 0.09 and 0.48 ± 0.05 mg/mL (Figure 6B), respectively, indicating that processed extracts had higher inhibition against both α-glucosidase and α-amylase than the raw sample (*p* < 0.05). The results indicated that the extracts of raw and processed PCH showed significant antidiabetic activity in vitro. After processing, the extracts exhibited a higher antidiabetic activity. However, further in vivo studies are needed.

## 3. Materials and Methods

### 3.1. Materials and Chemicals

LC/MS grade of methanol, acetonitrile and formic acid from Merck (Darmstadt, Germany) were purchased. Ultra-pure water was obtained from Watsons Food and Beverage Company (Guangzhou, China). Reference substances, including D-rhamnose (Rha), arabinose (Ara), D-mannose (Man), D-glucose (Glu), D-galactose (Gal) and D-fructose (Fru) from Chengdu Kangbang Biotechnology Co., Ltd. (Chengdu, China) were purchased. Diosgenin was obtained from the National Institute for the Control of Pharmaceutical and Biological Products (Beijing, China). Daidzein and scoparone were purchased from Chengdu Maide Biotechnology Co., Ltd. (Chengdu, China). Each compound was 98% purity as indicated by the supplier.

2,2-diphenyl-1-picrylhydrazyl (DPPH) and acarbose were supplied by Chengdu Kangbang Biotechnology Co. Ltd. (Chengdu, China). Trifluoroacetic acid, hydroxylamine hydrochloride, pyridine, acetic anhydride and trichloromethane from Chroma-Biotechnology Co., Ltd. (Chengdu, China) were purchased. 2,20-azinobis (3-ethylbenzothia-zo-line-6-sulfonicacid) diammonium salt (ABTS) was supplied by Beijing Soleibao Technology Co., Ltd. (Beijing, China). Phosphate buffer solution (PBS), 3,5-dinitrosalicylic acid (DNS) reagent, α-glucosidase and α-amylase were supplied by Shanghai Maclean Biochemical Technology Co., Ltd. (Shanghai, China). Ferric chloride (FeCl_3_), potassium ferricyanide (K_3_[Fe(CN)_6_]), trichloroacetic acid (Cl_3_CCOOH), potassium persulfate (K_2_S_2_O_8_), perchloric acid, anthrone, sulfuric acid, glacial acetic acid and soluble starch were supplied by Tianjin Zhiyuan Chemical Reagent Co., Ltd. (Tianjin, China). All other chemicals were analytically pure.

Black beans were obtained from the Chengdu Hehuachi medicinal herbs market. Ten batches of *Polygonatum cyrtonema* Hua samples were collected from Sichuan, Guangxi and Guangdong provinces in China and identified by Professor Yuan Liu (School of Pharmacy, Southwest Minzu University, China). All the samples were stored in the herbarium of the School of Pharmacy of Southwest Minzu University (Chengdu, China). Sample information is presented in Table 3.

### 3.2. Processing Methods of PCH

The raw PCH: The fresh samples were dried in a drying oven at 45 °C until complete dryness and then cut into thin slices. Processed PCH: Black beans (100 g) were boiled in a pot, and 1 L of juice was obtained. Raw PCH (10 kg) was soaked in 1 L of black bean juice for 12 h and then steamed for the outside, until the insides were moist black. Finally, samples were cut into thick slices and dried in a drying oven at 45 °C.

### 3.3. UHPLC-Q-Exactive-MS/MS Analysis

#### 3.3.1. Preparation of Sample Solution

All the batches of raw and processed PCH samples were ground to powder and sieved through a 10 mesh sieve. Then, the dried powder (1.0 g) was suspended in 25 mL of methanol, sonicated for 60 min at room temperature, filtered and dried. The residue was dissolved in methanol and fixed in a 5 mL volumetric flask. The extracted solution was filtered through a 0.22 μm microporous filters prior to UHPLC-Q-Exactive-MS/MS analysis.

#### 3.3.2. Ultra Performance Liquid Chromatography

The extracts were analysed by a Thermo Scientific™ Vanquish™ Flex UHPLC (Thermo Fisher Scientific Inc., Waltham, MA, USA) coupled with a binary gradient pump, automatic sampler, and diode array detector (DAD). Chromatographic separation was achieved using a Hypersil GOLD C18 column (2.1 × 100 mm, 1.8 μm). The mobile phases were 0.1% aqueous formic acid (A) and acetonitrile (B) with a flow rate of 0.3 mL/min. The optimized gradient elution conditions were set as follows: 0–3 min, 10–20% B; 3–8 min, 20% B; 8–10 min, 20–46% B; 10–15 min, 46% B; 15–20 min, 46–70% B; 20–25 min, 70–100% B. The column temperature was maintained at 30 °C and the injection volume was 5 μL.

#### 3.3.3. MS Conditions

After chromatographic separation, a Thermo UHPLC-Q-Exactive Orbitrap mass spectrometer was connected to the Thermo Scientific™ Vanquish™ Flex UHPLC system via a heating of the electrospray ionization (HESI) source for mass spectrometry. Positive ionization mode was employed to obtain data in range of *m*/*z* 100 to 1500 Da with 0.2 s scan time in a 30 min analysis period. The mass data was obtained under the following parameters: a source temperature of 100 °C and a desolvation temperature of 350 °C. The capillary voltage was 3.8 kV (positive mode); the sheath gas pressure was 3.5 MPa; the auxiliary gas pressure was 1.0 MPa, and the collision energy was 40 eV. Finally, Xcalibur 13.0 software (Thermo Fisher Scientific Inc., USA) was used to acquired and analysed the mass data.

### 3.4. Preparation and Determination of Polysaccharides Content

#### 3.4.1. Sample Preparation

The dried powder (100 g) was mixed with 1500 mL 80% ethanol and then sonicated for 1 h at 60 °C in water bath. The extraction step was repeated 3 times. The ultrasonic power was set at 250 W. The combined supernatant was filtered using a Buchner funnel and then concentrated with a rotary evaporator (Büchi R-100, Essen, Germany) under reduced pressure. To completely remove the solvent, the concentrated extracts further underwent lyophilization for 48 h using an FD-1B-80 lyophilizer (Boyikang, Beijing, China). Then, the PCH extracts were kept at 4 °C.

#### 3.4.2. Determination of Polysaccharides Content

The polysaccharide content of PCH was measured using an anthrone-sulfuric acid method described in the Chinese Pharmacopoeia 2020 Edition (Volume I) [19]. The dried PCH extracts were dissolved with purified water to the concentration of 1 mg/mL. An amount of 0.2 mL of extract solutions and 1.8 mL purified water was mixed in the test tube with a stopper. After that 8.0 mL of 2% anthrone-sulfuric acid was drawn slowly in an ice bath and shaken ten times. The test tube was then placed into a boiling water bath for heating 10 min and rapidly cooled down to the indoor temperature. The absorbance of 200 μL postreaction solutions was determined at a wavelength of 582 nm in 96-well plates using a Varioskan LUX2 automatic microplate reader (Thermo Fisher Scientific Inc., USA). For this, 0.2 mL deionized water replaced the extract solution as a blank. The results were calculated by using g of polysaccharides per 100 g of sample.

#### 3.4.3. Determination of Saponin Content

The saponin content of PCH was measured in the light of reports by Wang et al. [32], with a few modifications. In brief, the dried PCH extracts were dissolved with methanol to the concentration of 1 mg/mL. Aliquots of 0.4 mL were added to the test tube with a stopper and vaporized solvent at 80 °C in a water bath. After that, 0.2 mL 5% vanillin-glacial acetic acid was added to the test tube, followed by 0.5 mL perchloric acid in an ice bath. The solutions were vortexed and heated at 60 °C for 15 min, cooled in an ice bath for 2 min and finally, 4 mL of glacial acetic acid was added and incubated for 5 min. The absorbance of 200 μL postreaction solutions was determined at a wavelength of 452 nm in 96-well plates. For this, 0.4 mL methanol replaced the extract solution as a blank. The results were calculated by using g of saponin per 100 g of sample.

### 3.5. GC-QQQ-MSMS Analysis

#### 3.5.1. Hydrolysis of Extracts

Ten milligrams of PCH extracts from Section 3.4.1 and 2 mL of 2 mol/L trichloroacetic acid were mixed in a 10 mL ampoule bottle. After that, the extracts were hydrolysed for 3 h at normal temperature. The hydrolysates were cleaned at 40 °C by using a Termovap Sample Concentrator (Hangzhou Aosheng Instrument Co., LTD, Hangzhou, China) and kept at 4 °C.

#### 3.5.2. Derivatization of Saccharide

The hydrolysis products were mixed with 10 mg hydroxylamine hydrochloride and 0.5 mL pyridine and reacted for 30 min at 90 °C in a thermostat water bath. After that, the hydrolysis products were removed, cooled down to normal temperature and mixed with 1 mL acetic anhydride. After reacting for 30 min, the hydrolysis products were cooled down to normal temperature. The solvent was cleaned up at 50 °C by using a Termovap Sample Concentrator. The residue was dissolved in 4 mL trichloromethane. The solution was filtered through a 0.22 μm microporous filter before GC-MS analysis.

#### 3.5.3. Preparation of Standard Solutions

Derivatization of monosaccharide (rhamnose, arabinose, mannose, glucose, galactose, fructose) was prepared as described in Section 3.5.2, and the concentration of each monosaccharide standard stock solution was 2.5 mg/mL. Then, a series of appropriate concentration mixed standard solutions were achieved from the stock solution by adding an appropriate volume of chloroform. The mixed standard solutions were kept at 4 °C and filtered using a 0.22 μm microporous filter for further GC-MS analysis.

#### 3.5.4. Validation Method

The quantitative GC-MS a method was validated in terms of linearity, precision, repeatability, stability and recovery. The linear relationship was achieved by precisely injecting appropriate concentrations of monosaccharide standard stock solution into the GC-MS system to draw the linear equation, correlation coefficient and linearity range. The detection limit (LOD) and quantification limit (LOQ) were assessed on the basis of signal-to-noise ratios (S/N) of 3:1 and 10:1. The precision was achieved by determining six injections of the same standard solutions. The intraday and inter-day tests were used to evaluate repeatability. The intraday test was analysed by injecting the same solution for six times on the same day. The inter-day test was determined three times a day for three consecutive days. Stability test was evaluated by injecting the same solution at 0, 2, 4, 8, 12, 24 h, respectively. The test was achieved by adding the corresponding components of 80%, 100% and 120% into the sample to ensure recovery. Each level was evaluated for three times. The relative standard deviation (RSD) of relative retention time (RRT) of compound was calculated to analyze the precision, repeatability, stability and recovery of the method.

#### 3.5.5. GC-MS Conditions

GC-MS analysis was carried out using a Agilent7000D Triple Quadrupole GC/MS (Agilent Technologies, Santa Clara, CA, USA). The separations were performed using an HP-5 m capillary column (30 m × 0.25 mm, 0.25 μm). The temperature of GC injector was 250 °C, the carrier gas was helium (99.99% purity), the split ratio was set at 20:1, and the injection volume was set to 1 μL each time. The oven temperature programs were as follows: the initial temperature of 150 °C, then the temperature was increased to 200 °C at a rate of 8 °C/min (held for 1 min). The temperature was subsequently increased to 260 °C at a rate of 10 °C/min (held 1 min).

Mass spectrometry was carried out on an electrospray ionization (ESI) source. The ion source temperature and quadrupole temperature were set at 230 °C and 150 °C, respectively. The solvent delay was 3 min, and the scan range was *m*/*z* 30–600.

### 3.6. Determination of Antioxidant Capacity

A weighed sample of 10 g powder was sonicated with 25 mL of 80% ethanol for 60 min, repeated for three times. After that, the solution was filtered, concentrated and freeze-dried for standby application. For antioxidant activity assay, raw and processed solution was further diluted to a series concentration in the range of 0.1 to 1.5 mg/mL. The antioxidant activity of the raw and processed PCH samples was evaluated via DPPH assays, ABTS assay and FRAP assay.

#### 3.6.1. DPPH Assay

The DPPH activity was assessed based on the method reported by Blois [33] with the same modifications. For the DPPH assay, 1 mL sample solution was mixed with 1 mL of the DPPH solution (0.1 mmol/mL) in a test tube. After that, the mixed solutions were left in darkness and then incubated for 30 min. Finally, 200 μL mixed solutions were placed in 96-well plates, and the absorbance at 517 nm was determined. The DPPH radical scavenging activity was calculated as Equation (1):

(1) $$\mathrm{Scavenging}\;\mathrm{activity}=(1-\frac{A_{s}-A_{c}}{A_{b}})\times 100\%$$

where *A_s_* represented the absorbance of 1 mL sample extract with 1 mL DPPH solution; *A_c_* is 1 mL sample extract with 1 mL 70% ethanol; and *A_b_* is 1 mL 70% ethanol with 1 mL DPPH solution. The concentration of the radical content reduced by 50% was defined as the IC_50_ value. The IC_50_ values were measured by equation.

#### 3.6.2. Assay of ABTS

The ABTS activity was evaluated according to the method mentioned by Moon et al. [34] with some modifications. For the ABTS assay, ABTS (384.076 mg) and K_2_S_2_O_8_ (50.064 mg) were dissolved in water and fixed in a 100 mL volumetric flask. The configured solution was kept in the darkness at normal temperature for 12 h. Phosphate buffer solution (pH = 7.4) was used to diluted the configured ABTS^+^ solution, and then an absorbance of 0.70 ± 0.02 was determined at a wavelength of 734 nm for ABTS^+^ analysis. Then, 0.5 mL sample solution was added to 1.5 mL of ABTS^+^ solution. After reaction for 10 min in the darkness, 200 μL mixed solutions was put into 96-well plates and determined at a wavelength of 734 nm. The DPPH radical scavenging activity was converted by Equation (2):

(2) $$\mathrm{Scavenging}\;\mathrm{activity}\;(\%)=(1-\frac{A_{s}}{A_{0}})\times 100\%$$

where *A_s_* represents the absorbance of 0.5 mL sample solution plus1.5 mL ABTS^+^ solution; *A*_0_ represents 0.5 mL 70% ethanol plus 1.5 mL ABTS^+^ solution. The IC_50_ values were calculated using regression analysis.

#### 3.6.3. Assay of FRAP

The FRAP assay was determined as the procedure described by Moon et al. [34] with slight modifications. For analysis, 0.5 mL sample solution, 2.5 mL of phosphate buffer solution (pH = 6.6) and 2.5 mL of 1% K_3_[Fe(CN)_6_] were mixed in 10 mL test tube and reacted at 50 °C for 20 min. After that, 2.5 mL of 10% Cl_3_CCOOH was added and reacted at room temperature for 10 min. Finally, 200 μL of mixed solution was placed in a 96-well plate, and the absorbance was determined at 700 nm. The solution without sample was used as blank control.

All antioxidant assays (DPPH, ABTS, FRAP) were achieved by using Thermo Fisher Scientific Varioskan LUX2 automatic microplate reader (Thermo Fisher Scientific Inc., USA) equipment. All the tests were repeated three times, and the average mean was calculated.

### 3.7. Inhibition of Hypoglycemic Activity

The hypoglycemic activity was measured in vitro via the inhibition of α-glucosidase and α-amylase. The PCH extracts from Section 3.4.1 were diluted with purified water to concentrations of 0.1, 0.5, 1, 1.5, 2, 3, and 4 mg/mL.

#### 3.7.1. α-Glucosidase Inhibitory Assay

The inhibition of α-glucosidase assay was evaluated according to a previous method reported by Zaharudin et al. [35], with a few modifications. In brief, P-nitrophenol (PNPG) was set as a substrate in the test. A total of 10 μL of sample solution and 50 μL of phosphate buffer solutions (pH = 6.8) were mixed, and then 10 μL of 4 U/mL α-glucosidase enzyme was added as the sample group. Then, 60 μL of phosphate buffer solution (pH = 6.8) was mixed with 10 μL of sample solution as a sample control. Subsequently, 50 μL of phosphate buffer solution, 10 μL of α-glucosidase and 10 μL of distilled water were mixed as the enzyme group, and 60 μL of phosphate buffer solution was mixed with 10 μL of purified water as an enzyme control. All of the test reactions occurred at room temperature for 10 min, and then 50 μL of 5.0 mmol/L PNPG was added. The mixture was vortexed and reacted for 30 min at 37 °C. After that, the reaction was terminated by adding 100 μL 1 mol/L sodium carbonate solution. The absorbance value was recorded at a wavelength of 405 nm. The positive control was acarbose. The percent inhibitory was calculated as Equation (3):

(3) $$\mathrm{Inhibitory}\;(\%)=(1-\frac{OD\mathrm{sample}-OD\mathrm{sample}\;\mathrm{control}}{OD\mathrm{enzyme}\;-OD\mathrm{enzyme}\;\mathrm{control}})\times 100\%$$

#### 3.7.2. α-Amylase Inhibitory Assay

The inhibition of α-amylase was measured based on the method mentioned by Yonemoto et al. [36] with slight modifications. In brief, 30 μL of sample solution and 30 μL of 10 U/mL α-amylase were added to the test tube and incubated at 37 °C for 15 min. Next, 30 μL of 1% soluble starch solution was added and reacted for 15 min. Then, the reaction was terminated by adding 50 μL of DNS reagent, and boiling at 100 °C for 10 min, then cooling down to room temperature. After that, the reaction mixture was diluted by adding 840 μL of phosphate buffer solution (pH = 6.8). Finally, 200 μL of the mixture was placed in a 96-well plate, and the absorbance was recorded at a wavelength of 540 nm. The percent inhibition was calculated using Equation (4):

(4) $$\mathrm{Inhibitory}\;(\%)=(1-\frac{OD_{C}-OD_{D}}{{OD}_{A}-OD_{B}})\times 100\%$$

The positive control was acarbose. Where *OD_C_* is the absorbance of sample solution or acarbose; *OD_D_* is the absorbance of phosphate buffer solution instead of α-amylase; *OD_A_* is the absorbance of purified water replaced sample solution; and *OD_B_* is the absorbance of distilled water and phosphate buffer solution instead of sample solution and α-amylase, respectively. The IC_50_ values were calculated by using regression analysis.

All hypoglycemic activity tests (α-glucosidase and α-amylase) were determined on Thermo Fisher Scientific Varioskan LUX2 automatic microplate reader (Thermo Fisher Scientific Inc., USA) equipment. All tests were repeated in triplicate, and the results were averaged.

### 3.8. Statistical Analysis

UHPLC-Q-Orbitrap-MS data were converted into the excel format using Xcalibur 13.0 software to align retention time and peak integration. The peak area information of all tentatively assigned compounds was used to further multivariate statistical analysis containing cluster heatmap and principal components analysis (PCA) by using TBtools and Origin Pro 2022, respectively. All values were presented as the means ± standard errors (SE) of triplicates. One-way analysis of variance (ANOVA) from SPSS statistical software (version 20.0, SPSS Inc., Chicago, IL, USA) was employed to assess differences in mean values among groups. *p* < 0.05 was considered statistically significant.

## 4. Conclusions

In this study, we investigated and compared the chemical compositions and biological activities of raw and processed PCH with black beans. The results revealed significant differences in chemical composition, polysaccharide content, saponin content and biological activities between the two samples. The polysaccharide and saponin content in PCH processed with black beans were notably higher compared to the raw samples. The average values of polysaccharide and saponin content in the raw samples were 12.19% and 5.01%, respectively. However, the average values of polysaccharide and saponin content in the samples processed with black beans were 32.18% and 7.69%, respectively. Furthermore, bioactivity assays demonstrated that the processed PCH with black beans exhibited stronger antioxidant and antidiabetic activities compared to the raw material. The IC_50_ values for DPPH assay were 1.0 ± 0.12 mg/mL for the raw PCH and 0.65 ± 0.10 mg/mL for the processed PCH with black beans. Similarly, the IC_50_ values for ABTS were 0.65 ± 0.07 mg/mL and 0.25 ± 0.04 mg/mL for the raw and processed samples, respectively. In terms of α-glucosidase inhibitory activity, the IC_50_ values were 5.58 ± 0.22 mg/mL and 1.29 ± 0.12 mg/mL for the raw and processed PCH, respectively. For α-amylase inhibitory activity, the IC_50_ values were 0.80 ± 0.09 mg/mL and 0.48 ± 0.04 mg/mL for the raw and processed samples, respectively. These results suggest that processing PCH with black bean juice can alter its chemical composition, content, and subsequently impact its antioxidant and antidiabetic activities. Additionally, this study highlights the significance of black bean-processing for PCH and provides a foundation for its further development as a functional food. However, it is essential to conduct further in vivo studies to demonstrate the role of black bean-processing in PCH’s activities.

## Figures and Tables

**Table 1:** The results of UPLC-Q-Exactive-MS/MS identification of chemical constituents from raw PCH and processed PCH.

No.	*t*/min	Precursor Ion (*m*/*z*)	Error /ppm	Fragment Ions (*m*/*z*)	Molecular Formula	Identification	Raw PCH	Processed PCH
1	3.668	144.08156 [M + H]^+^	4.93	128.05013, 115.05492, 91.05509	C_10_H_9_N	6-Methylquinoline	+	+
2	4.109	169.07701 [M + H]^+^	−0.93	169.07664, 168.06664, 125.06008	C_8_H_9_FN_2_O	3-Fluoro-N’-hydroxy-4-methylbenzenecarboximidamide	+	+
3	4.130	328.11978 [M + H]^+^	1.7	135.08096, 131.04948, 121.06533, 105.07039, 103.05487	C_19_H_13_N_5_O	3-Methyl-6-oxo-1-phenyl-4-(3-pyridinyl)-6,7-dihydro-1H-pyrazolo[3,4-b]pyridine-5-carbonitrile	-	+
4	4.695	212.11839 [M + H]^+^	3.33	195.09239, 167.07089, 119.06101, 94.06588, 77.03954	C_13_H_13_N_3_	N,N’-Diphenylguanidine	+	+
5	5.639	198.12854 [M + H]^+^	4.13	181.10182, 166.07823, 106.06578, 91.05499, 79.05508	C_14_H_15_N	Dibenzylamine	+	+
6	5.643	433.11496 [M + H]^+^	4.29	271.06094, 243.06612, 215.07085, 153.01874, 91.05487	C_21_H_20_O_10_	unknow	-	+
7	7.662	319.082 [M + H]^+^	2.43	273.07654, 245.08148, 167.03447, 163.03957, 123.04462	C_16_H_14_O_7_	Padmatin	+	-
8	9.081	284.12912 [M + H]^+^	3.45	164.07121, 147.04465, 121.06541, 119.04980, 103.05490	C_17_H_17_NO_3_	Paprazine	+	+
9	10.059	255.06584 [M + H]^+^	2.53	227.07100, 199.07602, 153.07043, 137.02394, 119.04977	C_15_H_10_O_4_	Daidzein	-	+
10	10.986	303.08786 [M + H]^+^	4.41	257.08173, 229.08670, 167.03453, 163.03960, 135.04468,	C_16_H_14_O_6_	unknow	+	-
11	11.570	181.05023 [M + H]^+^	3.87	163.11285, 135.04454, 107.08614, 91.05480, 67.05514	C_9_H_8_O_4_	4-oxo-4,5,6,7-tetrahydrobenzo[b]furan-3-carboxylic acid	+	+
12	11.571	193.05038 [M + H]^+^	3.95	165.05515, 137.06024, 109.06550, 91.05497, 68.99794	C_10_H_8_O_4_	5,7-Dihydroxy-4-methylcoumarin	+	-
13	11.792	271.0614 [M + H]^+^	4.82	243.06604, 215.07108, 169.06546, 153.01881, 121.02882	C_15_H_10_O_5_	Genistein	-	+
14	12.214	295.22791 [M + H]^+^	3.44	227.21707, 151.11235, 135.11743, 95.08626, 67.05510	C_18_H_30_O_3_	13(S)-HOTrE	+	-
15	12.276	202.21744 [M + H]^+^	4.76	184.20668, 85.10194, 71.08640, 62.06094, 57.07082	C_12_H_27_NO	N,N-Dimethyldecylamine N-oxide	+	-
16	12.468	287.09293 [M + H]^+^	4.05	167.03462, 147.04471, 119. 04974, 91.05491, 68.99786	C_16_H_14_O_5_	unknow	+	-
17	12.631	211.08733 [M + H]^+^	3.51	193.07681, 192.06796, 165.07085, 115.05492, 105.07058	C_13_H_10_N_2_O	5,7-Dihydro-6H-dibenzo[d,f][1,3]diazepin-6-one	+	+
18	13.128	315.08737 [M + H]^+^	3.37	313.07111, 286.08279, 241.08656, 213.09215, 198.06728	C_17_H_14_O_6_	Aflatoxin B2	+	+
19	13.941	207.06589 [M + H]^+^	3.41	179.07083, 164.04680, 148.05188, 133.06537, 108.04921	C_11_H_10_O_4_	Scoparone	+	+
20	14.844	301.10815 [M + H]^+^	4.29	179.03517, 137.06004, 121.06537, 122.06859, 91.05498	C_17_H_16_O_5_	unknow	+	+
21	16.153	437.19495 [M + Na]^+^	3.48	119.08613, 117.07032, 91.05495, 79.05503	C_24_H_30_O_6_	Bis(4-ethylbenzylidene)sorbitol	+	-
22	18.692	219.17557 [M + H]^+^	4.22	201.16444, 163.11180, 135.08084, 123.11755, 81.07075	C_15_H_22_O	Nootkatone	+	-
23	19.537	320.25732 [M + H]^+^	−3.2	95.08607	C_20_H_33_NO_2_	unknow	+	+
24	20.040	235.16977 [M + H]^+^	2.26	179.10745, 180.11069, 123.04454, 57.07079	C_15_H_22_O_2_	3,5-di-tert-Butyl-4-hydroxybenzaldehyde	-	+
25	20.122	359.14987 [M + H]^+^	2.67	341.13843, 235.09723, 219.06676, 175.07613, 137.06026	C_20_H_22_O_6_	Matairesinol	+	+
26	20.372	279.23309 [M + H]^+^	4.77	137.13301, 123.11738, 109.10186, 95.08624, 93.07060	C_18_H_30_O_2_	α-Eleostearic acid	+	+
27	21.366	355.28586 [M + H]^+^	4.09	337.27499, 263.23770, 245.22723, 161.13303, 133.10173	C_21_H_38_O_4_	1-Linoleoyl glycerol	-	+
28	21.390	478.32339 [M + H]^+^	3.48	434.26038, 390.19824, 329.20343, 285.13789, 258.12930	C_33_H_39_N_3_	unknow	+	+
29	21.411	227.21759 [M + H]^+^	4.25	241.19543, 221.15463, 171.11710, 161.13290, 151.11226	C_18_H_30_O_3_	9-Oxo-10(E),12(E)-octadecadienoic acid	+	+
30	21.554	279.16013 [M + H]^+^	3.73	205.08685, 167.03441, 150.02721, 149.92388, 121.02893	C_16_H_22_O_4_	Dibutyl phthalate	+	+
31	21.829	284.33249 [M + H]^+^	4.63	60.08168, 57.07076	C_19_H_41_N	Cetrimonium	+	+
32	22.269	338.34314 [M + H]^+^	4.05	321.31607, 303.30536, 212.20132, 149.13286, 135.11731	C_22_H_43_NO	Erucamide	+	+
33	22.533	324.29108 [M + H]^+^	4.24	306.28064, 263.23862, 245.22665, 179.18025, 147.11760	C_20_H_37_NO_2_	Linoleoyl Ethanolamide	+	+
34	23.066	293.24863 [M + H]^+^	3.83	261.22220, 151.11247, 123.11769, 109.10181, 81.07066	C_19_H_32_O_2_	9(Z),11(E),13(E)-Octadecatrienoic Acid methyl ester	+	+
35	23.434	300.29102 [M + H]^+^	4.38	283.26355, 123.11771, 109.10162, 95.08624, 85.10188	C_18_H_37_NO_2_	Palmitoyl ethanolamide	+	+
36	23.873	326.30673 [M + H]^+^	4.23	309.27997, 135.11768, 121.10165, 83.08633	C_20_H_39_NO_2_	Oleoyl ethanolamide	+	+
37	23.891	311.16556 [M + H]^+^	4.47	255.10117, 203.10631, 177.05539, 161.09665, 135.04459	C_20_H_22_O_3_	Avobenzone	+	+
38	24.121	161.06029 [M + H]^+^	3.26	133.06534, 118.04193, 105.07047, 79.05498, 66.04727	C_10_H_10_O_3_	4-Methoxycinnamic acid	+	-
39	24.357	256.26447 [M + H]^+^	3.85	130.12366, 116.10772, 102.09198, 95.08604, 88.07630	C_16_H_33_NO	Hexadecanamide	+	+

**Table 2:** Polysaccharides and Saponin content and information of Sample.

No.	Collecting Location	Collection Year	Classification	Total Saccharide Content (%)	Total Saponin Content (%)
G1	Meishan, Sichuan	June 2020	Raw	8.46 ± 0.65	5.02 ± 0.42
G2	Meishan, Sichuan	June 2020	Raw	14.04 ± 1.07	5.97 ± 0.33
G3	Meishan, Sichuan	June 2020	Raw	8.54 ± 0.24	3.64 ± 0.65
G4	Mianyang, Sichuan	October 2020	Raw	14.88 ± 0.86	4.73 ± 0.49
G5	Mianyang, Sichuan	October 2020	Raw	13.45 ± 0.75	5.59 ± 0.55
G6	Baise, Guangxi	May 2021	Raw	14.59 ± 1.32	4.72 ± 0.63
G7	Baise, Guangxi	May 2021	Raw	10.84 ± 0.87	4.31 ± 0.61
G8	Baise, Guangxi	May 2021	Raw	10.35 ± 0.53	5.28 ± 0.73
G9	Heshan, Guangdong	June 2021	Raw	14.48 ± 1.16	5.22 ± 0.48
G10	Heshan, Guangdong	June 2021	Raw	12.25 ± 0.88	5.63 ± 0.61
P1	Meishan, Sichuan	June 2020	processed	32.12 ± 2.21	8.50 ± 0.46
P2	Meishan, Sichuan	June 2020	processed	32.27 ± 1.86	8.20 ± 0.50
P3	Meishan, Sichuan	June 2020	processed	29.40 ± 0.90	7.86 ± 0.62
P4	Mianyang, Sichuan	October 2020	processed	33.78 ± 1.69	9.41 ± 0.85
P5	Mianyang, Sichuan	October 2020	processed	35.12 ± 2.11	7.82 ± 0.46
P6	Baise, Guangxi	May 2021	processed	32.37 ± 2.30	6.09 ± 0.49
P7	Baise, Guangxi	May 2021	processed	32.02 ± 0.95	7.58 ± 0.82
P8	Baise, Guangxi	May 2021	processed	32.90 ± 0.70	6.71 ± 0.35
P9	Heshan, Guangdong	June 2021	processed	30.57 ± 1.06	6.63 ± 0.44
P10	Heshan, Guangdong	June 2021	processed	32.21 ± 0.82	8.09 ± 0.37

**Table 3:** The results of linear regression, LOQs and LODs.

Compound	Regression Equation	Linearity Range (μg/mL)	r	LOQ (μg/mL)	LOD (μg/mL)
rhamnose	*y* = 3615416637*x* − 5554691	2.528~25.275	0.9996	8.173 × 10^−1^	3.342 × 10^−1^
arabinose	*y* = 1777422855*x* − 2702648	5.065~50.650	0.9996	6.573 × 10^−1^	2.471 × 10^−1^
mannose	*y* = 1501991081*x* − 3075994	5.085~50.850	0.9995	5.438 × 10^−1^	4.184 × 10^−1^
glucose	*y* = 1051468840*x* − 2269974	25.225~504.500	0.9995	9.935 × 10^−1^	7.488 × 10^−1^
fructose	*y* = 357575280*x* − 1347648	25.450~254.500	0.9988	4.126	8.836 × 10^−1^

## Data Availability

Not applicable.
